# Parasymphyseal fracture location in lateral compression pelvic ring injuries: an assessment of reoperation and radiographic displacement in an international multicenter study

**DOI:** 10.1007/s00590-026-04896-7

**Published:** 2026-08-03

**Authors:** Wayne Hoskins, Charles Gusho, Rown Parola, Daniel Bravin, Brett Crist, Alyssa Wenzel, Jaime Bellamy, Kyle Schweser, Gregory Della Rocca, Josh Milby, James Stannard, Douglas Haase

**Affiliations:** 1 Department of Orthopaedic Surgery, Whangarei Hospital, Whangarei 0110, New Zealand; 2 University of Melbourne, Melbourne, Australia; 3 University of Missouri, Columbia, USA; 4 Cox Health Springfield, Springfield, USA; 5 University of Missouri–Kansas City, Kansas City, USA

**Keywords:** Lateral compression, Pelvic ring, Parasymphyseal fracture, Surgical fixation, Deformity, Reoperation

## Abstract

**Purpose:**

To compare reoperation rates and radiographic outcomes between parasymphyseal fracture location and those in extra-symphyseal zones for lateral compression (LC) pelvic ring injuries.

**Methods:**

This retrospective study included patients with acute, operatively treated LC injuries. Anterior ring fractures were classified based on a modified Nakatani classification as parasymphyseal (zone Ia), extra-symphyseal (zone Ib), zones II or III. Patient, fracture and implant characteristics were collected. The primary outcome was unplanned reoperation; secondary was radiographic loss of reduction. Comparisons were made across zones, with a sub-analysis restricted to LC1 injuries. Multivariable logistic regression was performed.

**Results:**

Among 275 LC injuries (mean age 59.8 ± 21.3 years; 56% [n = 155] female; reoperation rates were 6.5% (n = 18) overall and did not differ significantly across zones (Ia 12% [7/60], Ib 5.7% [3/53], II 6.8% [7/103], III 1.7% [1/59]; *p* = 0.2). Radiographic deformity differed significantly across zones (Ia 27% [16/60], Ib 23% [12/53], II 24% [25/103], III 3.4% [2/59]; *p* = 0.004), driven by the markedly lower rate in zone III. Multivariable regression confirmed deformity was associated with the low rate in zone III, while older age (OR 1.02 per year, 95% CI 1.00–1.04) and ramus comminution (OR 2.11, 95% CI 1.06–4.20) were independently associated with deformity. Reoperation did not differ by zone. When restricted to 183 LC1 injuries (mean age 64.1 ± 19.5 years; 61% [n = 112] female), findings were similar.

**Conclusion:**

Parasymphyseal fractures represent a challenging LC cohort. Loss of reduction is common but driven primarily by comminution and patient age rather than fracture location alone.

## Background

Lateral compression (LC) pelvic ring injuries represent a heterogenous spectrum of injury with varying patient symptoms, degrees of instability, risk of fracture displacement and associated morbidity [1–8]. Fracture location, pattern, bone quality and fracture classification systems influence stability and treatment outcomes [1, 5–7, 9–11]. While the indications for operative management of many Young-Burgess LC1 (LC1; AO/OTA 61-B1/B2) injuries remain debated [11], there is broader consensus for surgical stabilization in more unstable LC1 patterns, as well as LC2 and LC3 injuries [7, 12–14].

With increasing recognition of the anterior ring's role in overall pelvic stability, a heightened awareness to accurate identification, appropriate reduction, and adequate stabilization of this portion in LC pelvic ring injuries has occurred over the past several years. [5–7, 9, 15]. The Nakatani classification system divides superior pubic ramus fractures into three zones: zone I (medial to the obturator foramen), zone II (within the obturator foramen), and zone III (lateral to the obturator foramen) [3]. Parasymphyseal fractures, located immediately adjacent to the pubic symphysis, may represent a distinct and particularly challenging subset within a modified Nakatani zone I (zone Ia) [4]. These fractures are frequently associated with poorer bone quality, higher comminution, and technical difficulties in achieving reliable intramedullary implant purchase with standard percutaneous techniques [3, 15–18]. Under-reported in the literature, parasymphyseal fractures may represent a particularly unstable subset within the LC spectrum, potentially leading to higher rates of loss of reduction, deformity, and surgical complications. There is no consensus for the optimal constructs for these fractures.

The authors hypothesized that parasymphyseal fracture location in operatively treated LC injuries is associated with higher rates of reoperation and radiographic loss of reduction compared to extra-symphyseal locations. This study aimed in operatively treated LC injuries to [1] compare reoperation rates and radiographic outcomes between parasymphyseal and other fracture locations and [2] evaluate the impact of different anterior ring surgical constructs.

## Methods

This retrospective multicenter study was conducted at three centers (University of Missouri, Columbia, Missouri, USA; Cox Health, Springfield, Missouri, USA; and Whangarei Hospital, Northland, New Zealand). Patients were identified using CPT codes (27,216 and 27,217) for acute, operatively treated Young-Burgess LC pelvic ring injuries (AO/OTA 61-B1/B2) from January 2019 to January 2026.

Inclusion criteria were acute injuries (< 3 weeks old) with operative management which included posterior ring fixation, minimum three-month follow-up, and radiographic confirmation of healing. Exclusion criteria included non-acute presentations, pathological fractures, non-unions, and cases without posterior ring fixation.

**Fig. 1:**
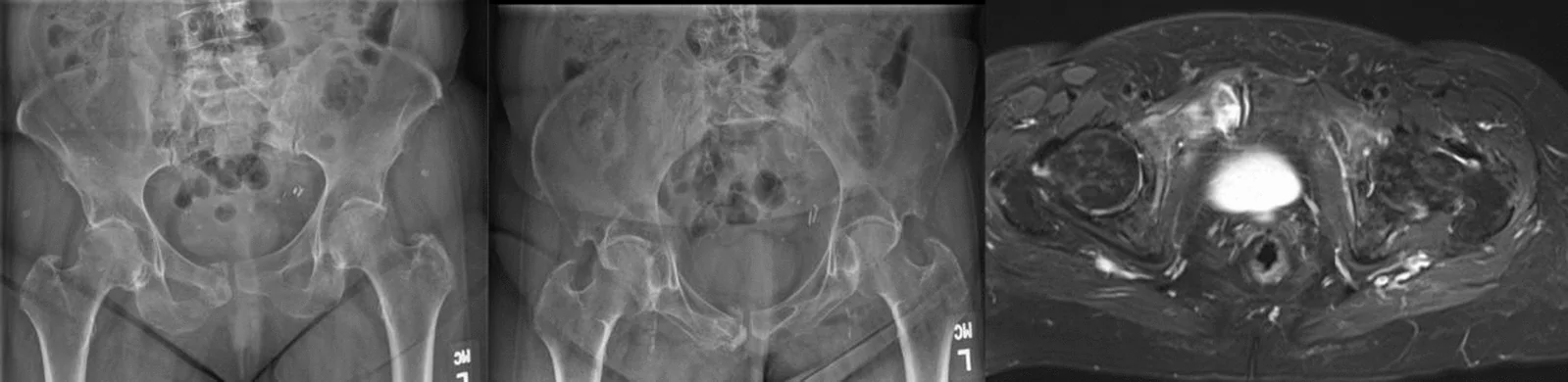
Example of a right sided parasymphyseal (zone Ia) fracture location

**Fig. 2:**
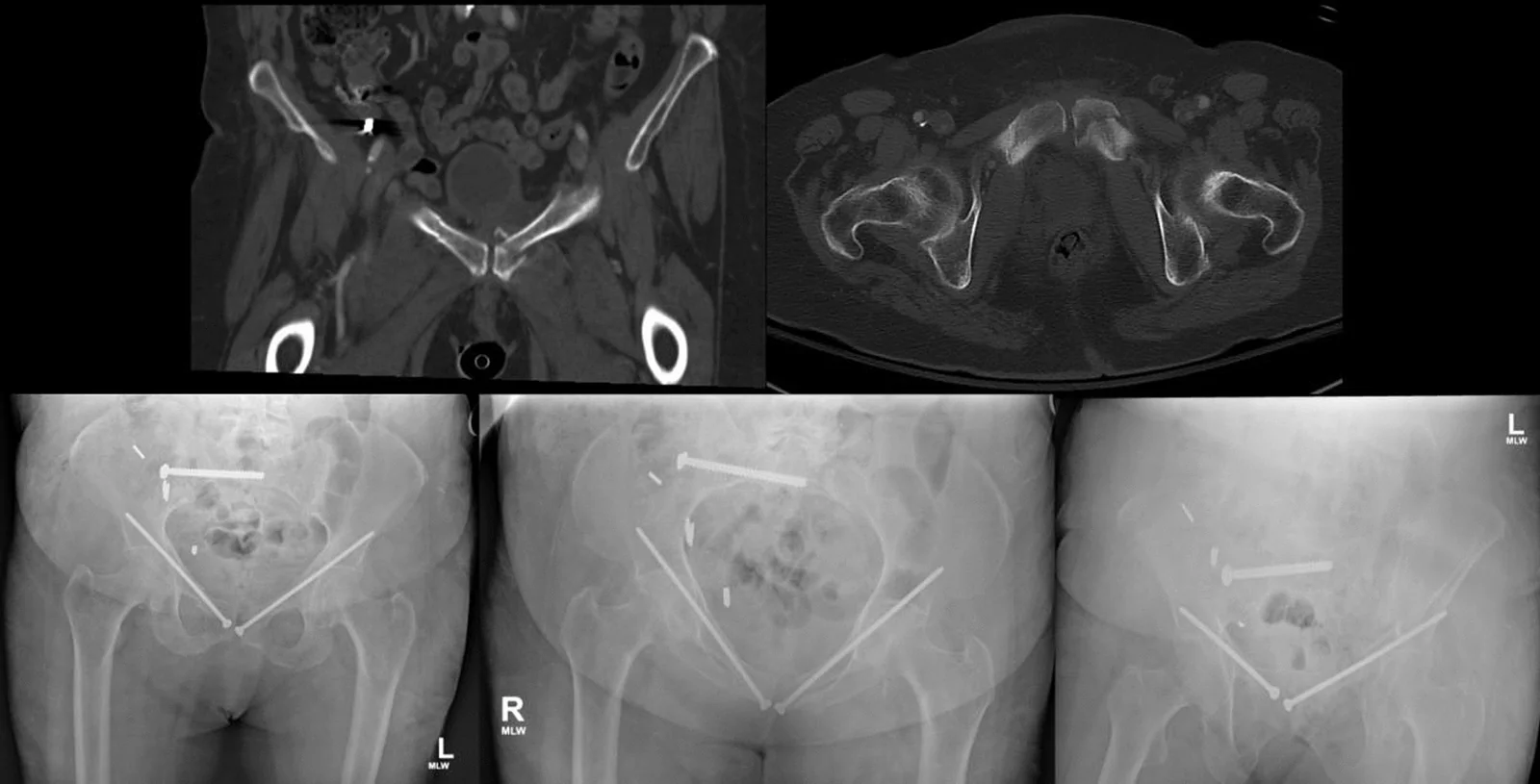
Example of a right sided zone 2 and left sided zone Ia fracture location, treated with retrograde bicortical medium diameter implants

Anterior ring fractures were classified based on the Nakatani Classification [3] as zone I, zone II, or zone III. Within Nakatani zone I (medial to the obturator foramen), fractures were subdivided into parasymphyseal (zone Ia), defined as fractures immediately adjacent to and abutting the pubic symphysis (Fig. 1), and extra-symphyseal (zone Ib), defined as the remainder of zone I that is medial to the obturator foramen but does not abut the symphysis. Additional variables included patient demographics, LC subtype, fracture comminution, displacement, angle, bilaterality, and the Mizzou Pelvic Displacement Predictor [MiPDiP] score [5]. The MiPDiP score is a radiographic instrument for predicting late displacement of LC1 injuries; it is derived from three independent radiographic predictors: superior ramus fracture angle (oblique [1 point]] or longitudinal [4 points]), superior ramus comminution (1 point), and initial anterior ring displacement (2 points), with higher scores indicating progressively greater risk of deformity [5]. Surgical details encompassed posterior ring fixation methods (iliosacral/trans-sacral implants, plates) and anterior ring fixation (none, retrograde/antegrade intramedullary implants [including cannulated screws, cannulated fasteners (Fasteners; OsteoCentric Technologies, Austin, TX) and curved intramedullary implants (CurvaFix; CurvaFix Inc, Bellevue, WA), InFix, Ex Fix, or plate). For intramedullary implants, length (long: bicortical or bypassing fracture by ≥ 2 Nakatani zones; short: otherwise) and diameter (small: 3.5–4.0 mm; medium: 4.5–5.0 mm; large: ≥ 6.5 mm) were also recorded  (Fig. 2).

The a priori primary outcome was unplanned reoperation related to the index injury (for loss of fixation, deformity correction, non-union repair, or symptomatic anterior ring implant removal). Planned InFix and Ex Fix removal were not counted as a reoperation. Planned or symptomatic removal of posterior ring implants in the absence of anterior ring fixation failure was not counted as a study reoperation. The secondary outcome was radiographic deformity, defined as greater than 1 cm of medial displacement between the postoperative and final healed radiographs [19]. Displacement was measured on standardized anteroposterior, inlet and outlet pelvic radiographs using the technique described by DeKeyser et al. [19], performed by trauma fellowship trained reviewers at each participating centre. We acknowledge that plain-radiograph measurements that are not corrected for radiographic magnification have limited reliability for detecting displacement [20]. Comparisons were performed by Modified Nakatani zone and, and among constructs, with LC1 sub-analysis.

### Statistical analysis

Statistical comparisons between groups were assessed using Pearson’s Chi-squared or Fisher’s exact test for categorical variables and Wilcoxon rank sum test for continuous variables. Data are reported as mean with standard deviation (SD) or as group percentages. Multivariable logistic regression was used to estimate the independent association of anterior ring fracture zone with each outcome, with zone III as the reference. The deformity model adjusted for age, sex, Young–Burgess LC subtype, ramus comminution and baseline ramus displacement. Because reoperation was infrequent and one zone contained a single event, the reoperation model used Firth penalized likelihood to reduce small-sample bias and avoid separation. Adjusted odds ratios are reported with 95% confidence intervals. Analyses were performed in R (version 4.x; packages stats and logistf; R Core Team [2024]. R Foundation for Statistical Computing, Vienna, Austria). *P* values < 0.05 were considered significant.

## Results

### All LC injuries

Of 275 included LC injuries the mean age was 59.8 ± 21.3 years; 56% (n = 155) were female; 67% (n = 183) were LC1; 15% (n = 42) LC2; and 18% (n = 50) LC3. Parasymphyseal fractures (zone Ia) comprised 22% (n = 60), Ib 19% (n = 53), zone II 37% (n = 103), and zone III 21% (n = 59).

Table 1 details demographics, fracture characteristics and fixation for LC injuries by modified Nakatani zone. Zone Ia and Ib fractures occurred in older patients (*p* < 0.001) more females (*p* < 0.018) and were more commonly LC1 patterns (*p* = 0.006). Zone Ia fractures showed greater comminution (*p* < 0.001). Zone III fractures were less likely to be displaced (*p* < 0.001), comminuted (*p* < 0.001) or longitudinal (*p* < 0.001). MiPDiP scores and fracture bilaterality were similar between all fracture locations (all *p* > 0.05). Anterior ring antegrade intramedullary fixation was most common (50%, n = 137), with higher rates in zone III (61%) and zone Ia (60%). Anterior ring retrograde intramedullary fixation was more frequent in zone Ib (30%). Long intramedullary implants were used in 84% of total cases, with lowest rates in zone II (73%, *p* = 0.030) and similar rates in zone Ia (89%), Ib (94%) and III (87%). Large diameter implants were used in 60% of cases with similar rates between all modified Nakatani zone. (*p* = 0.2).

**Table 1:** Demographics and radiographic and surgical outcomes of lateral-compression pelvic ring injuries by anterior ring modified nakatani zone fracture location

Characteristic	Overall, N = 275	Ia, N = 60	Ib, N = 53	II, N = 103	III, N = 59	*p*-value
Age (Years)	59.76 (21.30)	65.87 (19.93)	66.36 (15.13)	55.60 (23.38)	54.88 (20.97)	< 0.001
Sex						0.018
Female	155/275 (56%)	38/60 (63%)	36/53 (68%)	57/103 (55%)	24/59 (41%)	
Male	120/275 (44%)	22/60 (37%)	17/53 (32%)	46/103 (45%)	35/59 (59%)	
Young-burgess fracture type						0.006
LC1	183/275 (67%)	47/60 (78%)	43/53 (81%)	55/103 (53%)	38/59 (64%)	
LC2	42/275 (15%)	7/60 (12%)	4/53 (7.5%)	20/103 (19%)	11/59 (19%)	
LC3	50/275 (18%)	6/60 (10%)	6/53 (11%)	28/103 (27%)	10/59 (17%)	
Superior ramus fracture						< 0.001
Displaced	130/275 (47%)	36/60 (60%)	29/53 (55%)	55/103 (53%)	10/59 (17%)	
Undisplaced	145/275 (53%)	24/60 (40%)	24/53 (45%)	48/103 (47%)	49/59 (83%)	
Ramus comminution	126/275 (46%)	41/60 (68%)	24/53 (45%)	49/103 (48%)	12/59 (20%)	< 0.001
Fracture bilaterality						0.7
Bilateral	85/275 (31%)	22/60 (37%)	14/53 (26%)	32/103 (31%)	17/59 (29%)	
Unilateral	190/275 (69%)	38/60 (63%)	39/53 (74%)	71/103 (69%)	42/59 (71%)	
Ramus fracture angle						< 0.001
Longitudinal	69/275 (25%)	18/60 (30%)	19/53 (36%)	29/103 (28%)	3/59 (5.1%)	
Oblique	142/275 (52%)	28/60 (47%)	28/53 (53%)	57/103 (55%)	29/59 (49%)	
Transverse	64/275 (23%)	14/60 (23%)	6/53 (11%)	17/103 (17%)	27/59 (46%)	
*MiPDiP score*						
0	45/275 (16%)	4/60 (6.7%)	3/53 (5.7%)	13/103 (13%)	25/59 (42%)	
1	40/275 (15%)	5/60 (8.3%)	5/53 (9.4%)	13/103 (13%)	17/59 (29%)	
2	38/275 (14%)	10/60 (17%)	7/53 (13%)	17/103 (17%)	4/59 (6.8%)	
3	39/275 (14%)	10/60 (17%)	11/53 (21%)	14/103 (14%)	4/59 (6.8%)	
4	56/275 (20%)	16/60 (27%)	12/53 (23%)	21/103 (20%)	7/59 (12%)	
5	16/275 (5.8%)	4/60 (6.7%)	6/53 (11%)	5/103 (4.9%)	1/59 (1.7%)	
6	17/275 (6.2%)	3/60 (5.0%)	6/53 (11%)	8/103 (7.8%)	0/59 (0%)	
7	24/275 (8.7%)	8/60 (13%)	3/53 (5.7%)	12/103 (12%)	1/59 (1.7%)	
*Anterior ring fixation construct*						
Antegrade intramedullary implant	137/275 (50%)	36/60 (60%)	17/53 (32%)	48/103 (47%)	36/59 (61%)	
Combined	5/275 (1.8%)	0/60 (0%)	1/53 (1.9%)	3/103 (2.9%)	1/59 (1.7%)	
Ex-fix	4/275 (1.5%)	0/60 (0%)	1/53 (1.9%)	3/103 (2.9%)	0/59 (0%)	
Infix	25/275 (9.1%)	6/60 (10%)	4/53 (7.5%)	11/103 (11%)	4/59 (6.8%)	
None	51/275 (19%)	7/60 (12%)	13/53 (25%)	16/103 (16%)	15/59 (25%)	
Plate	8/275 (2.9%)	1/60 (1.7%)	1/53 (1.9%)	6/103 (5.8%)	0/59 (0%)	
Retrograde intramedullary implant	45/275 (16%)	10/60 (17%)	16/53 (30%)	16/103 (16%)	3/59 (5.1%)	
Anterior ring implant length						0.030
Long (bicortical)	153/182 (84%)	41/46 (89%)	31/33 (94%)	47/64 (73%)	34/39 (87%)	
Short (unicortical)	29/182 (16%)	5/46 (11%)	2/33 (6.1%)	17/64 (27%)	5/39 (13%)	
N/A (No fixation)	93	14	20	39	20	
*Anterior ring implant diameter*						0.2
Large	110/182 (60%)	30/46 (65%)	19/33 (58%)	34/64 (53%)	27/39 (69%)	
Medium	74/182 (41%)	17/46 (37%)	14/33 (42%)	32/64 (50%)	11/39 (278%)	
Small	1/182 (0.5%)	0/46 (0%)	0/33 (0%)	0/64 (0%)	1/39 (2.6%)	
N/A (No fixation)	93	14	20	39	20	

^1^ Mean (SD); n/N (%) . ^2^ Kruskal–Wallis rank sum test; Pearson’s Chi-squared test; Fisher’s exact test. ^*^MiPDiP—Mizzou pelvic displacement predictor

Table 2 reports outcomes. Reoperation rates were 6.5% (n = 18) overall and did not differ significantly across zones (Ia 12% [7/60], Ib 5.7% [3/53], II 6.8% [7/103], III 1.7% [1/59]; *p* = 0.2). Radiographic deformity differed significantly across zones (Ia 27% [16/60], Ib 23% [12/53], II 24% [25/103], III 3.4% [2/59]; *p* = 0.004), the difference being driven principally by the markedly lower rate in zone III.

**Table 2:** Outcomes of lateral compression injuries based on modified Nakatani zone fracture location

Characteristic	Overall, N = 275	Ia, N = 60	Ib, N = 53	II, N = 103	III, N = 59	*p*-value
Subsequent reoperation	18/275 (6.5%)	7/60 (12%)	3/53 (5.7%)	7/103 (6.8%)	1/59 (1.7%)	0.2
Deformity development	55/275 (20%)	16/60 (27%)	12/53 (23%)	25/103 (24%)	2/59 (3.4%)	0.004

^1^ Mean (SD); n/N (%). ^2^ Kruskal–Wallis rank sum test; Pearson’s Chi-squared test; Fisher’s exact test

After adjustment for age, sex, LC subtype, ramus comminution and baseline displacement, the difference in radiographic deformity across zones was driven by the low rate in zone III; zones Ia, Ib and II did not differ from one another (Ia vs Ib adjusted OR 0.99, 95% CI 0.40–2.43; Ia vs II OR 1.41, 0.63–3.18) (Table 3). Older age (OR 1.02 per year, 1.00–1.04) and ramus comminution (OR 2.11, 1.06–4.20) were independently associated with deformity. In the Firth-penalized model, reoperation did not differ significantly across zones (Ia vs III OR 5.04, 95% CI 0.84–30.22; *p* = 0.08) (Table 4). Among the 182 isolated intramedullary-screw constructs, deformity occurred in 5/153 (3.3%) long/bicortical versus 12/29 (41.4%) short/unicortical implants; five of the seven zone-Ia reoperations involved no anterior ring screw, indicating that construct selection, not zone alone, may account for the observed differences. A fully adjusted zone-by-construct model was precluded by the small number of events.

**Table 3:** Multivariable regression analysis for deformity (n = 275, 55 events)

Variable (ref)	OR	95% CI	*p*
Zone Ia (vs III)	4.88	0.97–24.71	0.055
Zone Ib (vs III)	4.83	0.96–24.26	0.056
Zone II (vs III)	6.89	1.50–31.55	0.013
Age (per year)	1.02	1.00–1.04	0.025
Female	1.28	0.65–2.55	0.476
LC2 (vs LC1)	1.31	0.53–3.24	0.555
LC3 (vs LC1)	0.52	0.18–1.49	0.222
Ramus comminution	2.11	1.06–4.20	0.033
Baseline displacement	1.40	0.71–2.74	0.330

Pairwise (reference = Ia): Ia vs Ib OR 0.99 (0.40–2.43), *p* = 0.98; Ia vs II OR 1.41 (0.63–3.18), *p* = 0.41; Ia vs III OR 0.20 (0.04–1.04), *p* = 0.055

**Table 4:** Multivariable regression analysis for reoperation–Firth penalized model (n = 275, 18 events)

Variable (ref)	OR	95% CI	*p*
Zone Ia (vs III)	5.04	0.84–30.22	0.077
Zone Ib (vs III)	2.40	0.34–16.94	0.378
Zone II (vs III)	2.77	0.48–16.08	0.255
Age (per year)	0.99	0.97–1.02	0.626
Female	1.83	0.66–5.10	0.244

### LC1 injuries

When restricted to the 183 LC1 injuries, the mean age was 64.1 ± 19.5 years and 61% (n = 112) were female. Zone Ia comprised 26% (n = 47), Ib 23% (n = 43), II 30% (n = 55) and III 21% (n = 38).

Table 5 details demographics, fracture characteristics and fixation for LC1 injuries by modified Nakatani zone. Zone III injuries occurred in younger patients (*p* = 0.003), in more males (*p* =  < 0.001), had less displacement (*p* < 0.001) and less longitudinal fractures (*p* < 0.001). Zone Ia, Ib, and II were similar, and all fracture locations had similar rates of bilaterality (all *p* > 0.05) and similar MiPDiP scores. Antegrade intramedullary implants were the most common anterior ring fixation (50%), but least common in zone Ib (30%), and highest in zone III (63%) and zone Ia (60%). Retrograde intramedullary implants were the next most common anterior ring fixation (20%), being most common in zone Ib (37%), followed by zone II (20%) and Ia (17%). Long intramedullary implants were used in 82%, with lowest rates in zone II (68%) and similar rates for zone Ia, Ib and III (84–93%) (*p* = 0.064). Large diameter implants were used in 63% with similar rates between all fracture locations (*p* = 0.5).

**Table 5:** Demographics and radiographic and surgical outcomes of lateral-compression type 1 pelvic ring injuries by anterior ring modified nakatani zone fracture location

Characteristic	Overall, N = 183	Ia, N = 47	Ib, N = 43	II, N = 55	III, N = 38	*p*-value
Age (Years)	64.13 (19.53)	69.23 (17.65)	67.21 (15.38)	63.55 (21.49)	55.16 (20.49)	0.003
Sex						< 0.001
Female	112/183 (61%)	33/47 (70%)	34/43 (79%)	31/55 (56%)	14/38 (37%)	
Male	71/183 (39%)	14/47 (30%)	9/43 (21%)	24/55 (44%)	24/38 (63%)	
Superior ramus fracture						< 0.001
Displaced	72/183 (39%)	27/47 (57%)	25/43 (58%)	18/55 (33%)	2/38 (5.3%)	
Undisplaced	111/183 (61%)	20/47 (43%)	18/43 (42%)	37/55 (67%)	36/38 (95%)	
Ramus comminution	75/183 (41%)	31/47 (66%)	17/43 (40%)	21/55 (38%)	6/38 (16%)	< 0.001
Fracture bilaterality						0.4
Bilateral	41/183 (22%)	14/47 (30%)	10/43 (23%)	9/55 (16%)	8/38 (21%)	
Unilateral	142/183 (78%)	33/47 (70%)	33/43 (77%)	46/55 (84%)	30/38 (79%)	
Ramus fracture angle						< 0.001
Longitudinal	42/183 (23%)	16/47 (34%)	16/43 (37%)	10/55 (18%)	0/38 (0%)	
Oblique	91/183 (50%)	21/47 (45%)	22/43 (51%)	33/55 (60%)	15/38 (39%)	
Transverse	50/183 (27%)	10/47 (21%)	5/43 (12%)	12/55 (22%)	23/38 (61%)	
*MiPDiP score*						
0	36/183 (20%)	4/47 (8.5%)	2/43 (4.7%)	9/55 (16%)	21/38 (55%)	
1	30/183 (16%)	3/47 (6.4%)	4/43 (9.3%)	11/55 (20%)	12/38 (32%)	
2	29/183 (16%)	8/47 (17%)	5/43 (12%)	13/55 (24%)	3/38 (7.9%)	
3	24/183 (13%)	7/47 (15%)	10/43 (23%)	6/55 (11%)	1/38 (2.6%)	
4	30/183 (16%)	12/47 (26%)	10/43 (23%)	8/55 (15%)	0/38 (0%)	
5	11/183 (6.0%)	3/47 (6.4%)	4/43 (9.3%)	4/55 (7.3%)	0/38 (0%)	
6	11/183 (6.0%)	3/47 (6.4%)	6/43 (14%)	2/55 (3.6%)	0/38 (0%)	
7	12/183 (6.6%)	7/47 (15%)	2/43 (4.7%)	2/55 (3.6%)	1/38 (2.6%)	
*Anterior ring fixation construct*						
Antegrade Intramedullary Implant	92/183 (50%)	28/47 (60%)	13/43 (30%)	27/55 (49%)	24/38 (63%)	
Ex-fix	2/183 (1.1%)	0/47 (0%)	0/43 (0%)	2/55 (3.6%)	0/38 (0%)	
Infix	13/183 (7.1%)	4/47 (8.5%)	2/43 (4.7%)	6/55 (11%)	1/38 (2.6%)	
None	39/183 (21%)	6/47 (13%)	12/43 (28%)	9/55 (16%)	12/38 (32%)	
Plate	1/183 (0.5%)	1/47 (2.1%)	0/43 (0%)	0/55 (0%)	0/38 (0%)	
Retrograde intramedullary implant	36/183 (20%)	8/47 (17%)	16/43 (37%)	11/55 (20%)	1/38 (2.6%)	
Anterior ring implant length						0.064
Long (bicortical)	105/128 (82%)	31/36 (86%)	27/29 (93%)	26/38 (68%)	21/25 (84%)	
Short (unicortical)	23/128 (18%)	5/36 (14%)	2/29 (6.9%)	12/38 (32%)	4/25 (16%)	
N/A (No fixation)	55	11	14	17	13	
Anterior ring implant diameter						0.5
Large	80/128 (63%)	22/36 (61%)	18/29 (62%)	22/38 (58%)	18/25 (72%)	
Medium	47/128 (37%)	14/36 (39%)	11/29 (38%)	16/38 (42%)	6/25 (24%)	
Small	1/128 (0.8%)	0/36 (0%)	0/29 (0%)	0/38 (0%)	1/25 (4.0%)	
N/A (No fixation)	55	11	14	17	13	

^1^ Mean (SD); n / N (%). ^2^ Kruskal–Wallis rank sum test; Pearson’s Chi-squared test; Fisher’s exact test. *MiPDiP—Mizzou pelvic displacement predictor

Table 6 reports outcomes. Among LC1 injuries, reoperation did not differ significantly across zones (Ia 11% [5/47], Ib 4.7% [2/43], II 5.5% [3/55], III 0% [0/38]; *p* = 0.2). Radiographic deformity differed significantly across zones (Ia 32% [15/47], Ib 21% [9/43], II 25% [14/55], III 2.6% [1/38]; *p* = 0.009), again driven principally by the lower rate in zone III.

**Table 6:** Outcomes of lateral compression type 1 injuries based on modified Nakatani zone fracture location

Characteristic	Overall, N = 183	Ia, N = 47	Ib, N = 43	II, N = 55	III, N = 38	*p*-value
Subsequent reoperation	10/183 (5.5%)	5/47 (11%)	2/43 (4.7%)	3/55 (5.5%)	0/38 (0%)	0.2
Deformity development	39/183 (21%)	15/47 (32%)	9/43 (21%)	14/55 (25%)	1/38 (2.6%)	0.009

^1^ Mean (SD); n / N (%). ^2^ Kruskal–Wallis rank sum test; Pearson’s Chi-squared test; Fisher’s exact test

## Discussion

LC pelvic ring injuries comprise a heterogeneous spectrum in which specific fracture patterns and radiographic features critically influence stability and outcomes. This multicenter study demonstrates that parasymphyseal fractures (modified Nakatani zone Ia) occur predominantly in older females, LC1 patterns and are associated with greater comminution; all features consistent with poorer bone quality. Radiographic loss of reduction differed significantly across anterior ring fracture zones (overall *p* = 0.004 for the full cohort and *p* = 0.009 for LC1 injuries), a difference driven principally by the markedly lower rate in zone III, with parasymphyseal (zone Ia) fractures among the highest (27–32%). Reoperation rates did not differ significantly across zones (11–12% in zone Ia; *p* = 0.2). Multivariable regression results support that patient age and comminution rather than parasymphyseal fracture location are the drivers of deformity development, with low event rates underpowering reoperation assessment. Deformity also appeared related to fixation with high rates when short and unicortical screws were used (41.1%), compared with long and bicortical screws (3.3%), and five of the seven zone-Ia reoperations involved InFix (not including planned removal) or no anterior fixation. This indicates that construct selection, rather than zone alone, may account for the observed differences. These findings align with prior descriptions of technical challenges in this region due to bone morphology and quality, as well as associations with occult instability in fragility fractures. Future research is required to improve outcomes in this challenging subgroup, including consideration for use of alternative implants or constructs to traditional percutaneous fixation.

This study’s findings extend prior work underscoring the unique challenges of parasymphyseal fractures. Starr et al. [3] first described difficulties with screw purchase in this region due to morphology and bone quality. More recently, parasymphyseal involvement has been linked to occult instability on stress radiographs in fragility fractures (74% vs 48%; *p* < 0.0001) [4] and receives higher points in a validated LC1 instability scoring systems owing to displacement propensity [8]. Literature is otherwise limited on parasymphyseal fracture location and in general has not been considered in most published literatures assessing outcomes of surgical management, or when comparing outcomes between operative and non-operative treatment [11]. The results of this study, suggest parasymphyseal fracture location is an important consideration for fracture instability and surgical outcomes. Improved reporting of parasymphyseal fracture location should be considered in future research.

**Fig. 3:**
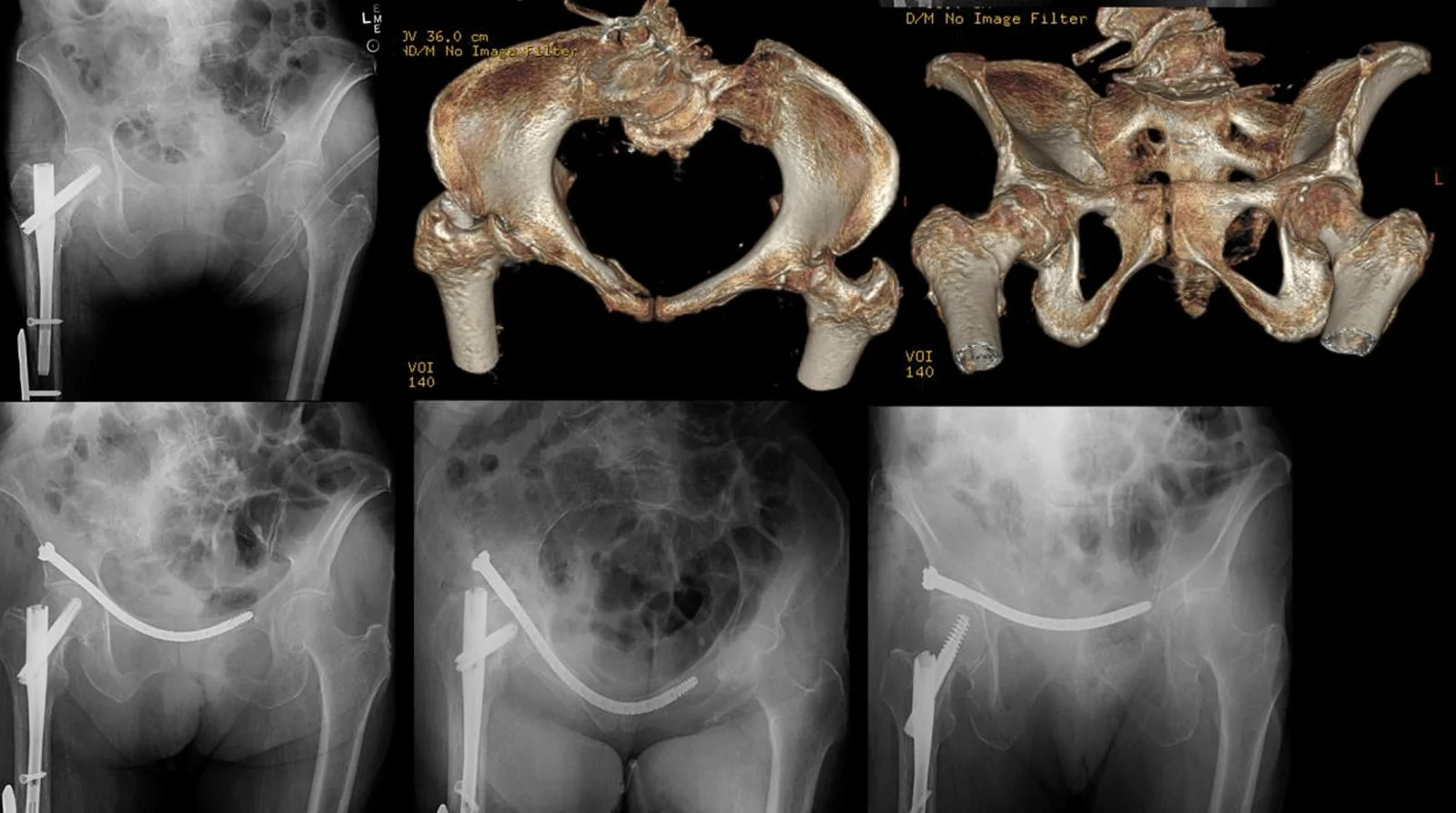
Example of a ‘smiley face’ construct using a curvafix implant

**Fig. 4:**
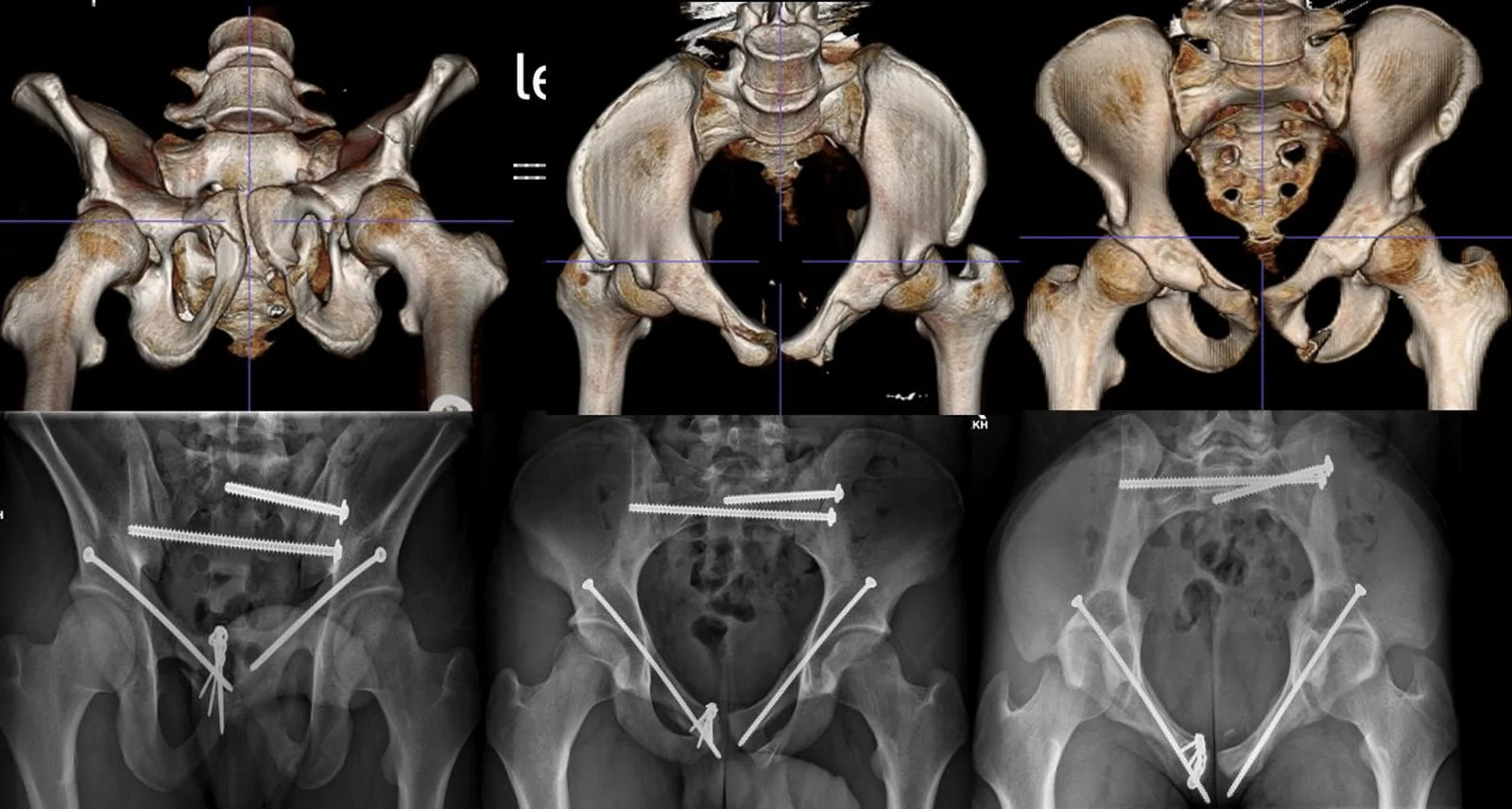
Example of an alternative construct for a parasymphyseal fracture location

Anterior ring fixation helps control displacement in LC injuries [5–7, 9, 15], yet failure can still persist in parasymphyseal cases. Failure likely occurs due to comminution, osteopenia, and limited intramedullary implant purchase near the symphysis. Loss of reduction and reoperation occurred even though long, large-diameter and bicortical intramedullary implants were frequently used; and results in the lowest deformity rates. Literature trends have favored bicortical fixation when treating the anterior ring [5–7, 9, 15], consistent with biomechanical data supporting superior stability [21, 22]. Adjunctive or alternative techniques, such as symphysis-crossing percutaneous implants [18] (Fig. 3) or anterior “W” plating [23], may warrant further investigation to reduce failure rates while balancing the morbidity of more invasive approaches [24]. In some circumstances, bicortical fixation may be impossible with percutaneous screws, and alternative techniques and constructs may be required (Fig. 4).

**Fig. 5:**
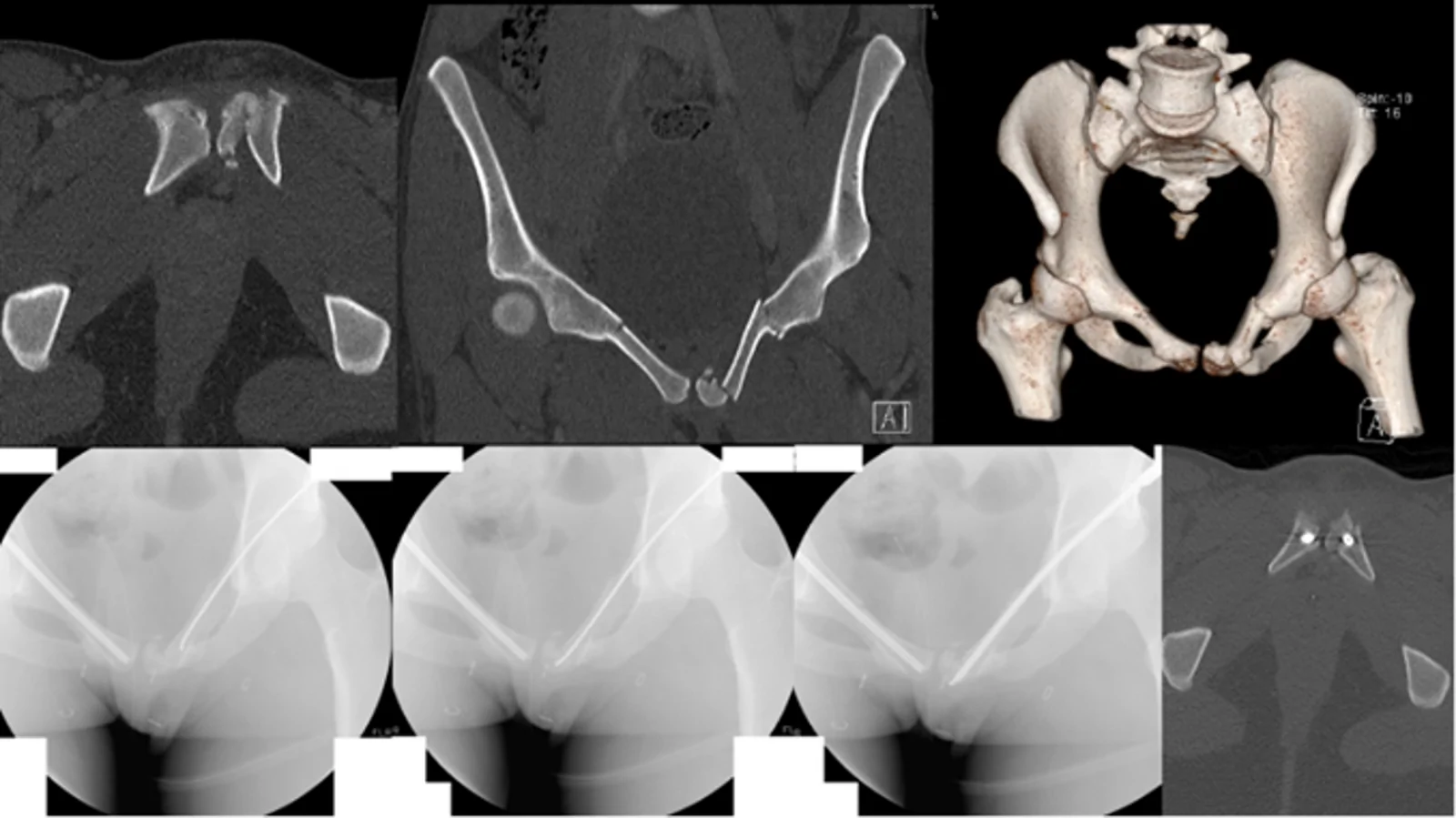
Example of a bent 2mm wire technique to allow exact finishing of an implant at the symphysis

The amount of antegrade intramedullary fixation compared to retrograde for zone 1a and zone 1b may appear surprising. It would seem easier to pass a retrograde implant from the small fracture fragment to the larger bony block. However, the fracture location and entry point required may compromise trajectory required to achieve bicortical purchase. Patient body habitus and composition can also impact a surgeon’s ability to safely perform percutaneous retrograde implant placement. Instead, antegrade techniques using bent 2mm wires are possible, which allow more control of where the implant will finish to allow for bicortical fixation (Fig. 5). Although retrograde rami screws, particularly short, unicortical constructs have been associated with higher rates of loss of fixation [15], we are not aware of clinical or biomechanical evidence directly comparing antegrade with retrograde bicortical intramedullary fixation. We therefore do not believe the antegrade-to-retrograde ratio explains the differences in radiographic and reoperation outcomes between zone Ia and Ib fractures, although a construct-stratified analysis would be required to exclude this.

This study has several limitations. The retrospective design introduces potential selection bias in the choice of fixation constructs. It is most likely that long and bicortical screws were not possible in the cases where they were not used. More importantly, the four zone groups differed substantially in age, sex, comminution, baseline displacement and fixation. Multivariable regression showed that age and comminution account for the observed differences in outcome, rather than fracture zone. The reoperations seen in zone Ia, for example, reflected specific implant choices, with most occurring with the use of InFix or no fixation, however we are underpowered to determine this. Larger studies are required for multivariable modelling using implant details. The moderate sample size for subgroup analysis risks type II error, and we did not perform multivariable analysis of the LC1 subanalysis. Patient reported and functional outcome data is also lacking. Finally there are reliability issues with radiographic measurements. Radiographic measurements may not be entirely reliable [25]. However, having a threshold of displacement above 1cm may help minimize that issue. Furthermore, 1cm of displacement, despite remaining an arbitrary figure, was chosen based on apparent acceptance as the threshold for instability and surgical management [19, 26] and it remains to be seen if there is a degree of displacement that correlates with clinical symptoms, function or reoperation risk.

## Conclusions

In operatively treated LC pelvic ring injuries, reoperations rates did not differ by anterior ring fracture zone. Loss of reduction was lowest in zone III and among the highest in parasymphyseal (zone Ia) fractures, whereas reoperation rates did not differ significantly. These associations are confounded by patient age and the presence of comminution as no difference in reoperation and deformity exists following multivariable regression. Higher rates of deformity occur when a long and bicortical screw is not used, however achieving this construct with this fracture pattern may not be possible. Surgeons should nonetheless remain vigilant when managing parasymphyseal fractures and consider optimized or alternative constructs to percutaneous fixation alone. Further biomechanical and clinical research is required to improve outcomes in this challenging subgroup. Prospective studies are needed to confirm these associations and define optimal fixation strategies for parasymphyseal LC injuries.

## Data Availability

No datasets were generated or analysed during the current study.

## References

[1] O’Hara NN, Sim D, Moore D et al (2022) Prospective characterization of pain and function in patients with unstable pelvic fractures treated with posterior screw fixation. J Orthop Trauma 36(11):557–563 10.1097/BOT.0000000000002416 35605147

[2] Mullis BH, Agel J, Jones C et al (2022) Unilateral sacral fractures demonstrate slow recovery of patient-reported outcomes irrespective of treatment. J Orthop Trauma 36(4):179–183 10.1097/BOT.0000000000002260 34483321

[3] Starr AJ, Nakatani T, Reinert CM et al (2008) Superior pubic ramus fractures fixed with percutaneous screws: what predicts fixation failure? J Orthop Trauma 22(2):81–87 10.1097/BOT.0b013e318162ab6e 18349774

[4] Patterson JT, Parry JA (2024) Lateral compression fragility fractures of the pelvis: diagnosis, classifications, and modern management. Curr Osteoporos Rep 22(6):621–631 10.1007/s11914-024-00891-1 PMC11499407 39313717

[5] Hoskins W, Parola R, Gusho C et al (2025) A predictive scoring system for late displacement and deformity following non-operative treatment of Young-Burgess lateral compression type 1 (OTA 61–B1/B2) pelvic ring injuries. Injury 56(10):112670 10.1016/j.injury.2025.112670 40795795

[6] Hoskins W, Gusho C, Bravin D et al (2025) Is there a gold standard for addressing the anterior ring in surgical fixation of Young-Burgess lateral compression type 1 (LC1; AO/OTA 61–B1/B2) pelvic ring injuries? Injury 56(12):112818 10.1016/j.injury.2025.112818 41108797

[7] Hoskins W, Gusho C, Haase D et al (2025) A comparison of anterior ring fixation constructs in Young-Burgess lateral compression type 2 and 3 (LC2, LC3; AO/OTA 61-B2/B3) pelvic ring injuries: does fixation matter? Injury 56(6):112320 10.1016/j.injury.2025.112320 40198968

[8] Beckmann J, Haller JM, Beebe M et al (2020) Validated radiographic scoring system for lateral compression type 1 pelvis fractures. J Orthop Trauma 34(2):70–76 10.1097/BOT.0000000000001639 PMC6982580 31524667

[9] Kumaran P, Wier J, Hasegawa I, Patterson JT, Gary JL (2024) Stability before and after percutaneous anterior medullary fixation of lateral compression 1 and 2 pelvic ring disruptions: should surgeons prioritize the anterior ring? Eur J Orthop Surg Traumatol 34(6):3103–3108 10.1007/s00590-024-04037-y 38965132

[10] Slobogean GP, Gaski GE, Nascone J et al (2021) A prospective clinical trial comparing surgical fixation versus nonoperative management of minimally displaced complete lateral compression pelvis fractures. J Orthop Trauma 35(11):592–598 10.1097/BOT.0000000000002088 33993178

[11] Orthopaedic Trauma Research Group (2026) Operative versus nonoperative treatment of stress-positive lateral compression type 1 pelvic ring injuries: a multicenter retrospective propensity-matched analysis. J Orthop Trauma 40(3):113–118 10.1097/BOT.0000000000003108 41182893

[12] Vallier HA, Lowe JA, Agel J et al (2019) Surgery for unilateral sacral fractures: are the indications clear? J Orthop Trauma 33(12):619–625 10.1097/BOT.0000000000001587 31425312

[13] Swenson RA, Paull TZ, Yates RA et al (2024) Comparison of operative and non-operative management of elderly fragility pelvic ring fractures. J Orthop Trauma. 10.1097/BOT.0000000000002863 39016440

[14] Booth A, Ingoe HMA, Northgraves M et al (2019) Effectiveness of surgical fixation for lateral compression type one (LC-1) fragility fractures of the pelvis: a systematic review. BMJ Open 9(5):e024737 10.1136/bmjopen-2018-024737 PMC6530388 31110085

[15] Tucker NJ, Stacey S, Kim YJ et al (2024) Variables associated with loss of fixation of retrograde rami screws in minimally displaced lateral compression type 1 pelvic ring injuries. J Orthop Trauma 38(4):215–219 10.1097/BOT.0000000000002756 38176888

[16] Varma JR, Foxall-Smith M, Donovan RL et al (2022) Surgical versus non-surgical treatment of unstable lateral compression type I (LC1) injuries of the pelvis with complete sacral fractures in non-fragility fracture patients: a systematic review. Cureus 14(9):e29239 10.7759/cureus.29239 PMC9573782 36262937

[17] Walker JB, Mitchell SM, Karr SD et al (2018) Percutaneous transiliac-transsacral screw fixation of sacral fragility fractures improves pain, ambulation, and rate of disposition to home. J Orthop Trauma 32(9):452–456 10.1097/BOT.0000000000001243 29916895

[18] Quercetti NF, DiStefano S, Yayac M, Getchell J, Ng MK (2026) Trans-symphyseal fixation for complex anterior pelvic fractures: a novel technique guide and case series. OTA Int 9(2):e479 10.1097/OI9.0000000000000479 PMC12999140 41859492

[19] DeKeyser GJ, Kellam PJ, Haller JM et al (2022) Emergency department stress radiographs of lateral compression type-1 pelvic ring injuries are safe, effective, and reliable. J Bone Joint Surg Am 104(4):e13 10.2106/JBJS.21.00737 34921551

[20] Orthopaedic Trauma Research (OTR) Group (2024) Fracture displacement of lateral compression type 1 (LC1) pelvic ring injuries: which measurement methods are reliable and does displacement correlate with adverse events? Eur J Orthop Surg Traumatol 34(7):3553–3559 10.1007/s00590-023-03776-8 37991594

[21] Hempen EC, Wheatley BM, Schimoler PJ et al (2022) A biomechanical comparison of superior ramus plating versus intramedullary screw fixation for unstable lateral compression pelvic ring injuries. Injury 53(12):3899–3903 10.1016/j.injury.2022.09.027 36182593

[22] McLachlin S, Lesieur M, Stephen D et al (2018) Biomechanical analysis of anterior ring fixation of the ramus in type C pelvis fractures. Eur J Trauma Emerg Surg 44(2):185–190 10.1007/s00068-017-0788-4 28391395

[23] Mauffrey C, David G, Fratus A, Krieg B, Onodera K, Tucker NJ (2025) The W-plate: a novel technique for fixation of unstable pelvic ring injuries. J Orthop Trauma 39(12):e73–e82 10.1097/BOT.0000000000003063 40892976

[24] Li P et al (2024) Percutaneous hollow nail internal fixation treatment for fractures of the pubic symphysis and its adjacent areas. Front Surg 11:1400834 10.3389/fsurg.2024.1400834 PMC11554617 39534695

[25] Lefaivre KA, Blachut PA, Starr AJ et al (2014) Radiographic displacement in pelvic ring disruption: reliability of 3 previously described measurement techniques. J Orthop Trauma 28(3):160–166 10.1097/BOT.0b013e31829efcc5 23760181

[26] Sagi HC, Coniglione FM, Stanford JH (2011) Examination under anesthetic for occult pelvic ring instability. J Orthop Trauma 25:529–536 10.1097/BOT.0b013e31822b02ae 21857421

