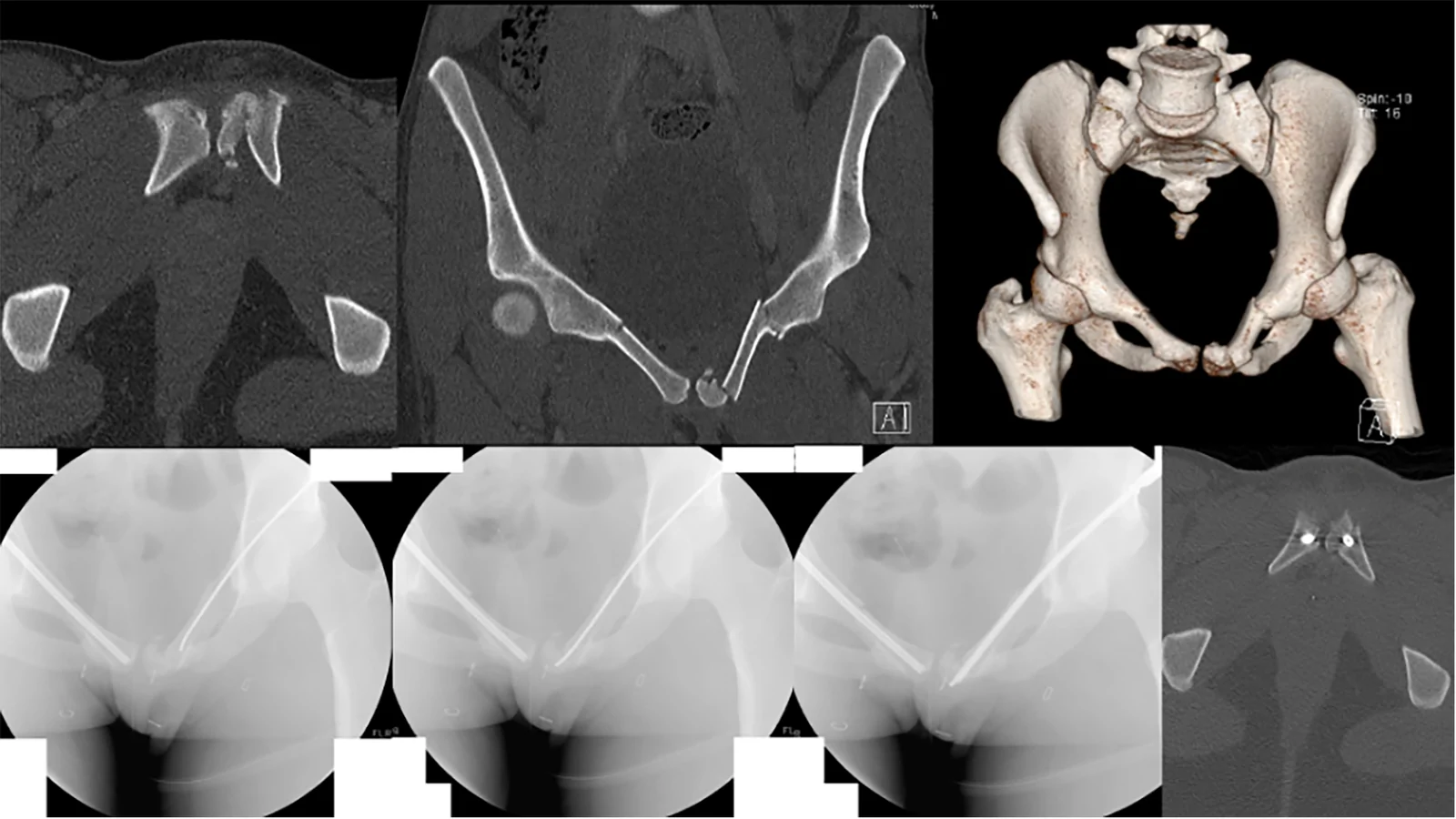# Correction: Parasymphyseal fracture location in lateral compression pelvic ring injuries: an assessment of reoperation and radiographic displacement in an international multicenter study

**DOI:** 10.1007/s00590-026-04964-y

**Published:** 2026-10-06

**Authors:** Wayne Hoskins, Charles Gusho, Rown Parola, Daniel Bravin, Brett Crist, Alyssa Wenzel, Jaime Bellamy, Kyle Schweser, Gregory Della Rocca, Josh Milby, James Stannard, Douglas Haase

**Affiliations:** 1 Department of Orthopaedic Surgery, Whangarei Hospital, Whangarei, 0110 New Zealand; 2 University of Melbourne, Melbourne, Australia; 3 University of Missouri, Columbia, USA; 4 Cox Health Springfield, Springfield, USA; 5 University of Missouri–Kansas City, Kansas City, USA

**Correction to: European Journal of Orthopaedic Surgery & Traumatology (2026) 36:317** **https://doi.org/10.1007/s00590-026-04896-7**

In the original version of this article the author’s name Gregory Della Rocca was incorrectly written as Gregory J. Della Rocca And also Figure 5 was appeared incorrectly and have now been corrected in the original publication. For completeness and transparency, the old incorrect versions are displayed below.

**Figure:**
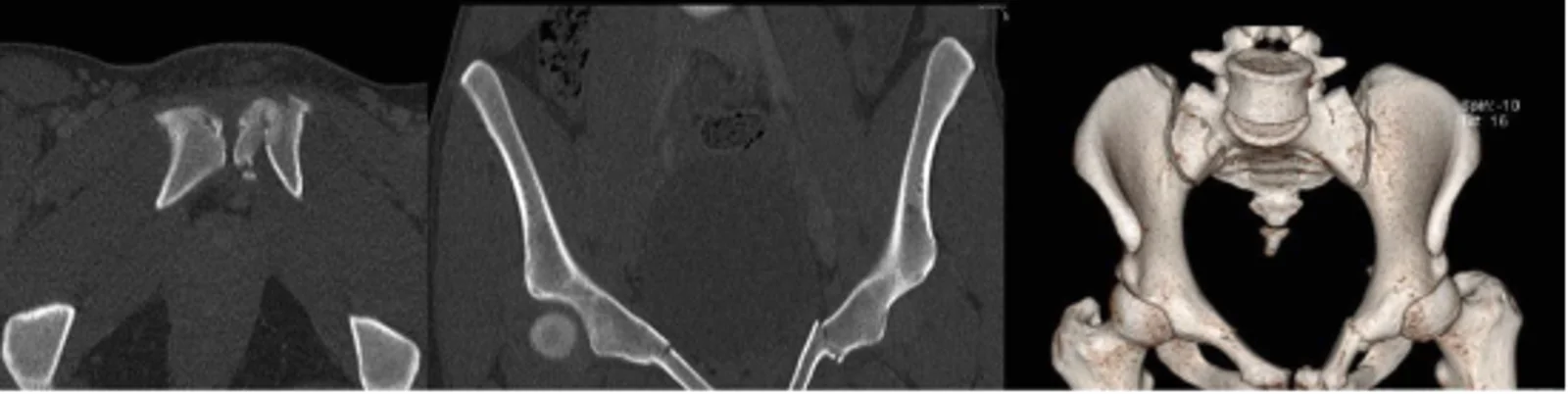

The original article has been corrected.

**Figure:**